# Mild Electrical Stimulation with Heat Shock Ameliorates Insulin Resistance via Enhanced Insulin Signaling

**DOI:** 10.1371/journal.pone.0004068

**Published:** 2008-12-30

**Authors:** Saori Morino, Tatsuya Kondo, Kazunari Sasaki, Hironori Adachi, Mary Ann Suico, Erika Sekimoto, Tomoko Matsuda, Tsuyoshi Shuto, Eiichi Araki, Hirofumi Kai

**Affiliations:** 1 Department of Molecular Medicine, Faculty of Medical and Pharmaceutical Sciences, Global COE “Cell Fate Regulation Research and Education Unit”, Kumamoto University, Kumamoto, Japan; 2 Department of Metabolic Medicine, Faculty of Medical and Pharmaceutical Sciences, Kumamoto University, Kumamoto, Japan; University of Bremen, Germany

## Abstract

Low-intensity electrical current (or mild electrical stimulation; MES) influences signal transduction and activates phosphatidylinositol-3 kinase (PI3K)/Akt pathway. Because insulin resistance is characterized by a marked reduction in insulin-stimulated PI3K-mediated activation of Akt, we asked whether MES could increase Akt phosphorylation and ameliorate insulin resistance. In addition, it was also previously reported that heat shock protein 72 (Hsp72) alleviates hyperglycemia. Thus, we applied MES in combination with heat shock (HS) to *in vitro* and *in vivo* models of insulin resistance. Here we show that 10-min treatment with MES at 5 V (0.1 ms pulse duration) together with HS at 42°C increased the phosphorylation of insulin signaling molecules such as insulin receptor substrate (IRS) and Akt in HepG2 cells maintained in high-glucose medium. MES (12 V)+mild HS treatment of high fat-fed mice also increased the phosphorylation of insulin receptor β subunit (IRβ) and Akt in mice liver. In high fat-fed mice and db/db mice, MES+HS treatment for 10 min applied twice a week for 12–15 weeks significantly decreased fasting blood glucose and insulin levels and improved insulin sensitivity. The treated mice showed significantly lower weight of visceral and subcutaneous fat, a markedly improved fatty liver and decreased size of adipocytes. Our findings indicated that the combination of MES and HS alleviated insulin resistance and improved fat metabolism in diabetes mouse models, in part, by enhancing the insulin signaling pathway.

## Introduction

It has been established that direct-current electrical fields impact on cellular functions [1]. Positive medical effects of applied low electric current, such as decreased inflammation, bone-fracture healing and alleviation of pain have been reported [2], [3], [4]. Low-intensity electric fields have also been shown to inhibit tumor growth *in vitro* [5] and *in vivo* with no serious side effects [6]. It is hypothesized that the therapeutic effects of applied low electrical field strength are due to enhanced signal transduction [7], a hypothesis that was partly validated by a study demonstrating that electrical signals promote wound healing through the activation of phosphatidylinositol-3-OH kinase (PI3 kinase) and Akt [8].

Insulin resistance, which characterizes type 2 diabetes, is manifested by decreased insulin-stimulated glucose transport and metabolism [9], [10]. This functional defect is partly due to a decrease in insulin-stimulated Akt activation and failure in the translocation of glucose transporter GLUT4 to the cell surface [11], [12], [13]. There is consensus that a marked reduction in insulin-stimulated PI3K-mediated activation of Akt results in decreased insulin sensitivity *in vivo* [14], [15], [16], [17]. Thus, enhancing Akt phosphorylation could alleviate insulin resistance.

Further studies have elucidated that insulin resistance could also be attributed to the serine phosphorylation of IRS-1, which is mediated by the activity of c-Jun N-terminal kinase (JNK) [18], [19]. It is now known that the activation of JNK is prevented by cellular protective actions of Hsp72 [20], [21], [22] and this implies a possible role of Hsp72 in ameliorating insulin resistance (reviewed in [23]). A recent report suggests that Hsp72 overexpression improved insulin resistance in high fat-fed mice [24]. Therefore Hsp72, which can be up-regulated by HS, may have an essential role in preventing insulin resistance.

In this study, we assessed the effects of heat shock (HS) together with mild electrical stimulation (MES) on insulin resistance in cellular and animal models. HS was produced by infrared rays, and low-intensity direct electrical current was delivered through insulated electrodes. Our results showed that HS+MES increased the insulin-stimulated phosphorylation of Akt in HepG2 cells maintained in high-glucose medium, which we used here as an *in vitro* model of insulin resistance. Moreover, HS+MES improved the hyperglycemic phenotype and fat metabolism in high fat-fed mice.

## Materials and Methods

### Antibodies

The antibodies used in this study were: mouse anti-Hsp72 (SPA-810), rabbit anti-Hsp72 (SPA-812) and rabbit anti-calnexin (C-terminus specific; SPA-860), from Stressgen Biotechnologies (Victoria, BC, Canada); mouse anti-c-Myc (9E10; sc-40), rabbit anti-IRS-1 (sc-559), and rabbit anti-phospho insulin receptor β-subunit (Tyr 1162/1163; sc-25103), from Santa Cruz Biotechnology (Santa Cruz, CA); rabbit antibodies from Cell Signaling Technology (Danvers, MA): anti-phospho-Akt (Ser 473), anti-Akt, anti-phospho-JNK (Thr183/Tyr185), anti-JNK and anti-phospho-(Tyr) p85PI3K binding motif.

### Cell culture and *in vitro* HS+MES treatment

**Figure 1:**
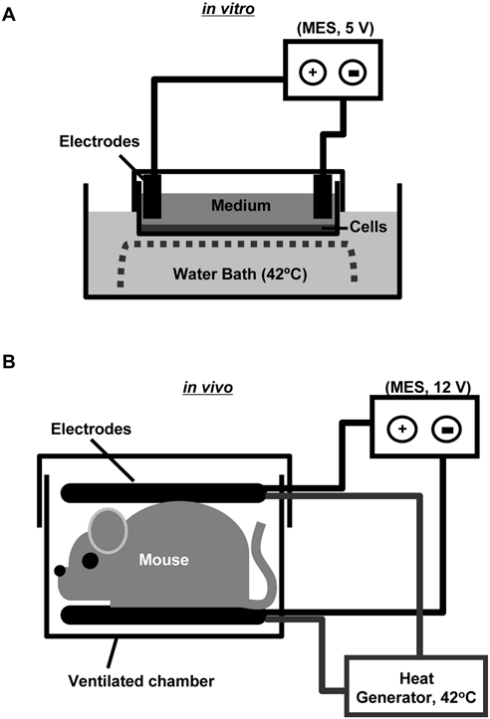
Diagram of apparatus for HS+MES treatment. (A) HS+MES treatment set-up for HepG2 cells. (B) HS+MES treatment set-up for mice.

Human hepatocyte cell line, HepG2 cells were maintained in Dulbecco's modified Eagle's medium (DMEM) containing 10% fetal bovine serum and antibiotics. Cells were plated on 60-mm culture dishes, and at 80% confluency were treated with HS and/or MES. For MES, the culture plate cover was exchanged with a plate cover slit at the sides designed to accommodate insulated wires bearing a pair of flat rubber electrodes, which were fitted at the walls of the culture plate and in contact with the culture media (Fig. 1A). The electrodes were connected to a Biometronome™ (Tsuchiya Gum Co., Ltd., Kumamoto, Japan). Electrical stimulation for cells was delivered using 5 V (55 pulses per second (pps)) of direct current with individual pulse duration of 0.1 ms [25]. The culture plate with the electrodes was carefully sealed and immersed in water bath at a temperature of 42°C. Simultaneous treatment with HS and MES was carried out for 10 min. After treatment, the medium was changed and cells were re-incubated at 37°C until assay. For control, cells were sham treated by setting up the electrodes in a similar manner as described above but without HS+MES.

### Animals and *in vivo* HS+MES treatment

Five-week-old male C57BL/6J mice and db/db mice (BKS.Cg-m+/+Leprdb/J: Leprdb/Leprdb mice; Charles River Laboratories, Inc., Kanagawa, Japan), were housed in a vivarium in accordance with the guidelines of the animal facility center of Kumamoto University. The mice were maintained on food (standard or high-fat diet) and water ad libitum. High-fat diet was composed of 14% lard, 14% beef tallow, 25% casein, 20% sucrose, 15% cornstarch, 5% cellulose. Six-week-old or 25-week-old mice were treated with HS and/or MES. A well-ventilated 12 cm×10 cm (width×height) chamber was designed for the animal treatment. Electrical stimulation was delivered to un-anesthetized test animal through a pair of 10-cm diameter electro-conductive and thermo-generative rubber electrodes, which were padded with moist soft cotton cloth. The electrodes can be adjusted to allow contact with the animal (Fig. 1B). The electrodes are connected to a Biometronome™ (Tsuchiya Gum Co., Ltd.) that delivered 12 V (55 pps) of direct current with individual pulse duration of 0.1 ms. The temperature at the surface of the electrodes was adjusted to 42°C. For the control group, mice were sham treated by putting them in the chamber with similar set-up as above for 10 min per session but without conducting HS+MES. All procedures involving these animals were approved by the Animal Care and Use Committee of Kumamoto University (#A19-115).

### Induction of insulin resistance in HepG2 cells

HepG2 cells were incubated in serum-free DMEM containing normal concentrations of glucose (5.5 mM D-glucose) for 16 h and were additionally incubated in serum-free DMEM containing either normal or high concentrations of glucose (25 mM D-glucose) for 24 h. During HS and/or MES treatment, cells were kept under normal or high glucose concentration. Cells were incubated with 100 nM insulin for 10 min prior to protein isolation.

### Immunoblotting

Protein lysates from HepG2 cells were isolated with radioimmunoprecipitation assay (RIPA) buffer [26] or lysis buffer (25 mM HEPES, 10 mM Na_4_P_2_O_7_·10H_2_O, 100 mM NaF, 5 mM EDTA, 2 mM Na_3_VO_4_, 1% Triton X-100). Mice tissues were lysed and sonicated in glycerol buffer [27]. Protein lysates were subjected to SDS-PAGE and Western blotting in the same manner as described previously [27]. The blots were probed with the indicated antibodies and their respective HRP-conjugated secondary antibodies obtained from Jackson ImmunoResearch Laboratories (West Grove, PA). The proteins were reacted with chemiluminescence reagent ECL (Amersham Pharmacia Biotech, UK) for visualizing the blots.

### Immunofluorescent staining

HepG2 cells on cover glasses were incubated and then fixed with 10% formalin. PIP3 and GLUT4 expressions were analyzed by immunohistochemistry in the same manner as described previously [28]. After PBS equilibration, slides or liver tissue sections were incubated with 3% normal goat serum in 2.5% Triton X-100/PBS for 1 or 2 h at room temperature for blocking. Incubation with a primary antibody to PIP3 (Echelon Biosciences Inc., Salt Lake City, UT, USA; 1/100 dilution) was performed overnight in blocking solution at 4°C. After washes with PBS, slides or sections were incubated with Alexa Fluor 546 or Alexa Fluor 488 (Molecular Probes, OR, USA), and DAPI (Dojindo, Kumamoto, Japan) solution in blocking solution for 2 h at room temperature. After three washes with PBS, slides or sections were mounted with ProLong Antifade kit (Molecular Probes) and examined with fluorescent microscope, BZ-8000 (KEYENCE, Osaka, Japan).

### Hsp72 plasmid, siRNA and transfection

A full-length cDNA for human Hsp72 (Hsp70A1A) was obtained using primers: 5′-primer, CTAGGATCCGTGTTCCGTTTCCAG; and 3′-primer, GACGAATTCCTCAATGGTGGGGCCT. The PCR product was cloned into pCR2.1 vector using a TA cloning kit (Invitrogen, Carlsbard, CA, USA). Hsp72 tagged with c-Myc in the C-terminus was constructed by subcloning Hsp72 into the *BamH*I-*EcoR*I sites of pCMV-Tag5 vector (Stratagene, La Jolla, CA, USA). Transient transfection of plasmid DNA was performed for 48 h using HilyMax (Dojindo, Kumamoto, Japan) according to the manufacturer's recommendations. Hsp72 siRNA was designed; sense, 5′-GGAGCUGGAGCAGGUGUGUTT-3′ and anti-sense, 5′-ACACACCUGCUCCAGCUCCTT-3′. GL2 (Luciferase) siRNA duplex was used as control for siRNA transfection; sense, 5′-CGUACGCGGAAUACUUCGATT-3′ and anti-sense, 5′-UCGAAGUAUUCCGCGUACGTT-3′. Transient transfection of siRNA duplex was performed for 24 h using TransIT-TKO (Mirus, Madison, WI, USA) following the manufacturer's protocol.

### Blood analysis and HOMA-IR index

Blood glucose was assayed with ACCU-CHEK Compact (Roche Diagnostics, Mannheim, Germany). Serum insulin, adiponectin and TNF-α were determined with LipoSEARCH (Skylight Biotech Inc., Akita, Japan). HOMA-IR was calculated using the following formula: fasting blood glucose×fasting insulin/22.5 [29].

### Glucose and insulin tolerance tests

Glucose tolerance test was performed on fasted (15 hr) mice. Mice received an injection of glucose (2 g/kg body wt), and blood glucose was assayed immediately before and at the indicated time after glucose administration. Insulin tolerance test was performed on fasted (6 h) mice. Mice received an injection of human regular insulin (2 units/kg body wt; Novo Nordisk, Copenhagen, Denmark) into the intraperitoneal space, and blood glucose was assayed immediately before and at the indicated time after injection.

### RT-PCR

Total RNA was collected from mice brown adipose tissue using Isogen (Nippongene, Tokyo, Japan). RT-PCR experiments were performed with an RT-PCR kit (Takara, Ohtsu, Japan) according to the manufacturer's instructions. The following primers were used for UCP1: up, 5′-TTTGGAAAGGGACGACCCCTAATC-3′, and down, 5′-ACCCGAGTCGCAGAAAAGAAGC-3′; for HPRT: up, 5′-GTTGGATACAGGCCAGACTTTGTTG-3′, and down, 5′-GATTCAACTTGCGCTCATCTTAGGC-3′.

### Statistical analysis

Data are presented as mean±S.E. Significance of the difference between groups were assessed with Student's unpaired two-tailed t test (when 2 groups were analyzed) or one-way ANOVA (for ≥3 groups). A *P* value of <0.05 was considered statistically significant.

## Results

### Treatment with HS+MES ameliorated hyperglycemia in high fat-fed mice

**Figure 2:**
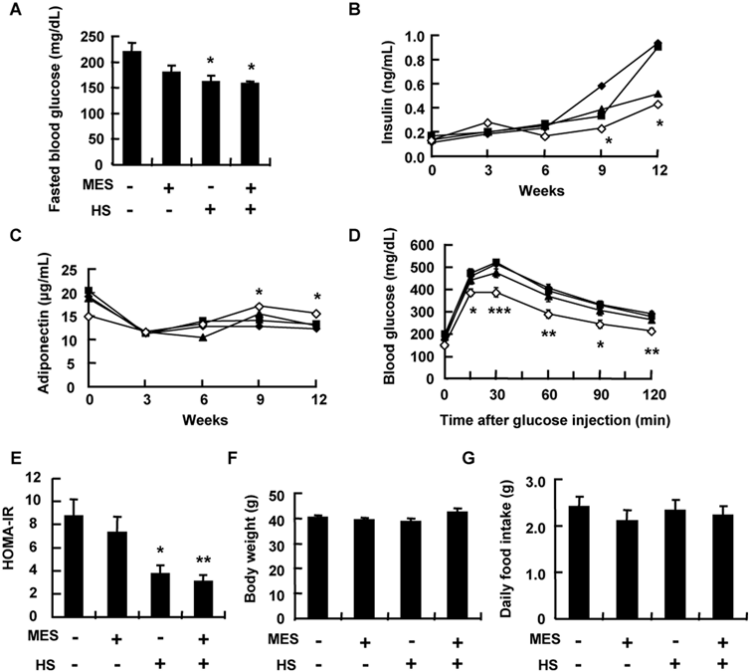
HS+MES ameliorated hyperglycemia in high fat-fed mice. (A) Blood glucose levels of high fat-fed sham-treated, HS and/or MES-treated mice were measured at the 12^th^ week of treatment in the 15-h fasted state. (B and C) Serum insulin (B) and adiponectin (C) of high fat-fed control, HS and/or MES-treated mice were measured every 3 weeks in the 15-h fasted state. (D) Intraperitoneal glucose tolerance test was performed on mice at the 12^th^ week of treatment in the 15-h fasted state. Glucose levels were measured at the time points indicated after glucose administration. Symbols: black diamonds = control; squares = MES; triangles = HS; white diamonds = HS+MES. (E) HOMA-IR was calculated as follows: fasting glucose×fasting insulin/22.5. Comparison was performed at 12^th^ week of treatment. (F and G) Body weight (F) and food intake (G) of high fat-fed control, HS and/or MES-treated mice were measured at the 12^th^ week of treatment. All data are presented as mean±S.E. (n = 6 per group) *P<0.05, **P<0.01, ***P<0.001 vs. control group, assessed by one-way ANOVA.

We assessed the effect of HS+MES treatment on hyperglycemia *in vivo* using high fat-fed mice. Five-week old male C57BL/6J mice were fed with a high-fat diet. One week after starting the high-fat diet, mice were sham-treated or treated with HS+MES for 10 min twice a week for 12–15 weeks. A low current (12 V; 0.1 ms) was well tolerated and did not cause pain or annoyance behavior in mice as observed by their tendency to stay still and occasionally doze off during treatment. After 12 weeks of treatment, we measured various blood parameters. The fasted blood glucose level was significantly lower in high fat-fed mice treated with HS+MES (Fig. 2A). Although the fasted blood glucose was also decreased in HS-treated group (Fig. 2A), the fasted insulin level in this group was not significantly lower compared with sham-treated group (Fig. 2B). On the other hand, the fasted insulin level was reduced significantly in HS+MES-treated mice after 9 to 12 weeks of treatment compared with control mice (Fig. 2B). At a similar time point, the HS+MES-treated mice had significant increase of adiponectin (Fig. 2C). HS+MES markedly improved glucose tolerance in high fat-fed mice as indicated by intraperitoneal glucose tolerance test (Fig. 2D). Homeostasis model assessment of insulin resistance, HOMA-IR index was also significantly improved in HS+MES-treated mice (Fig. 2E). Body weight and food intake did not differ between the control and treated groups (Fig. 2F,G).

### Treatment with HS+MES ameliorated hyperglycemia in mice chronically fed with high-fat diet

**Figure 3:**
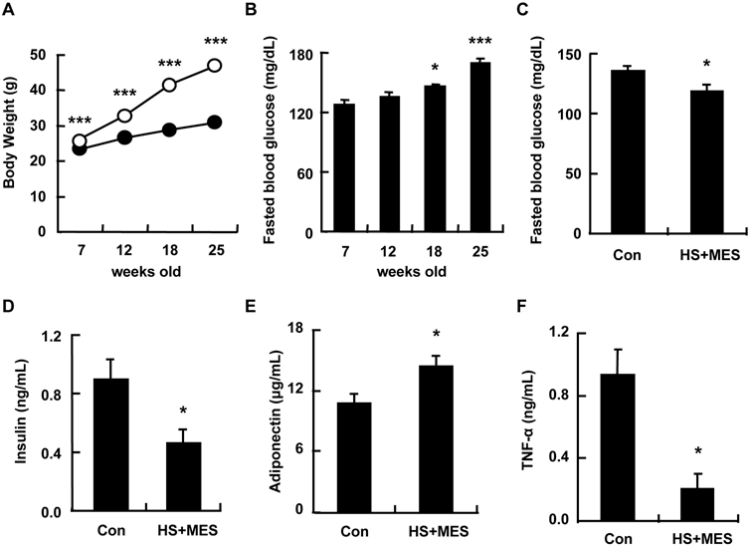
HS+MES ameliorated the diabetic phenotype in mice chronically fed with high-fat diet. (A) Six-week old C57BL/6J mice were fed with standard or high-fat diet for 5 months, and body weight was measured at the indicated time points. Symbols: black circles = standard diet; white circles = high-fat diet. Data are presented as mean±S.E. (n = 10 per group) ***P<0.001 assessed by unpaired *t* test. (B) Fasting blood glucose levels of mice fed with high fat-diet were measured at the indicated time points. Data are presented as mean±S.E. *P<0.05, ***P<0.001 vs. 7-week old mice, assessed by one-way ANOVA. (C–F) Five months after the start of high-fat feeding, mice were sham-treated (control) or treated with HS+MES for 10 min twice a week for 10 weeks. Blood glucose (C), serum insulin (D), adiponectin (E) and TNF-α (F) of control or HS+MES-treated high fat-fed mice (n = 5 per group) were measured at the 10^th^ week of treatment in the 15-h fasted state. Data are presented as mean±S.E. *P<0.05 assessed by unpaired *t* test.

We next examined the effect of HS+MES on mice fed with high-fat diet for 5 months. Body weight of high fat-fed mice was remarkably higher than those fed a standard diet (Fig. 3A). Moreover, the fasting blood glucose level of these high fat-fed mice significantly increased after 3–5 months of feeding on high-fat diet (18–25 weeks old vs 7 weeks old; Fig. 3B), indicating an onset of obesity and hyperglycemia. To determine whether HS+MES ameliorates hyperglycemia in these high fat-fed mice, we sham treated or treated this group of mice with HS+MES for 10 min twice a week for 10 weeks. HS+MES significantly decreased fasting blood glucose and insulin levels, and increased serum adiponectin (Fig. 3C–3E). Interestingly, HS+MES also markedly reduced the level of tumor necrosis factor-α (TNF-α), which is known to attenuate insulin signaling [30], in high fat-fed mice (Fig. 3F). These data collectively suggest that HS+MES treatment ameliorated hyperglycemic phenotype in mice chronically fed with high-fat diet.

### Treatment with HS+MES increased the insulin-stimulated phosphorylation of IRβ and Akt in the liver of high fat-fed mice

**Figure 4:**
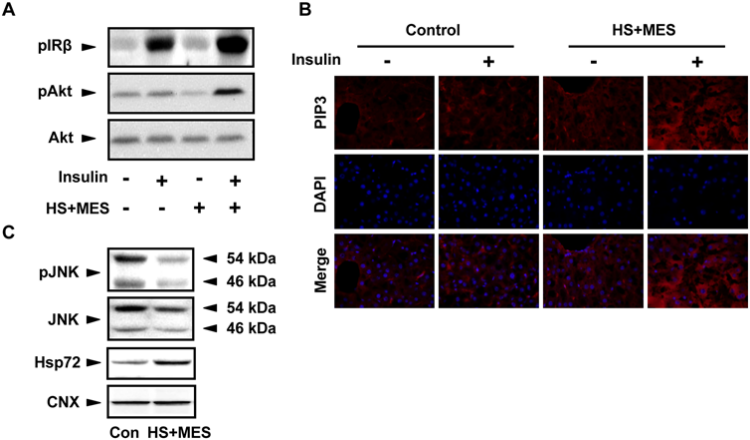
HS+MES improved insulin signaling in the liver of high fat-fed mice. (A) Liver lysates were extracted 15 weeks after initiation of HS+MES treatment from high fat-fed control and HS+MES-treated mice with or without 5 units of insulin stimulation through inferior vena cava, and were analyzed by Western blotting. (B) Liver tissues were isolated at the 15^th^ week after initiation of treatment from high fat-fed control or HS+MES-treated mice with or without 5 units of insulin stimulation through inferior vena cava. Tissues were dissected in frozen sections, stained with PIP3 and DAPI, and visualized by fluorescent microscope. Scale bars, 100 µm. (C) Liver lysates extracted from control and HS+MES-treated mice were subjected to Western blotting analysis using the indicated antibodies.

The results above suggest that HS+MES might have improved the insulin signaling in high fat-fed mice. To confirm whether HS+MES promotes insulin receptor phosphorylation, which initiates insulin action by phosphorylating multiple intracellular substrates, we determined the phosphorylation status of IRβ in mice liver. Lysates were extracted from the liver of sham-treated or HS+MES-treated high fat-fed mice 15 weeks after the start of treatment. Insulin administration, as expected, increased the IRβ phosphorylation (Fig. 4A, lane 2). Interestingly, HS+MES treatment enhanced the insulin-stimulated IRβ phosphorylation (Fig. 4A, pIRβ, lane 4), and activated IRβ downstream molecule, Akt (Fig. 4A, pAkt, lane 4). Consistent with these results, HS+MES enhanced the immunoreactivity of phosphatidylinositol-3,4,5-triphosphate (PIP3), a second messenger molecule produced by PI3K activation in mice liver (Fig. 4B). Moreover, JNK phosphorylation was substantially down-regulated in liver lysates of HS+MES-treated mice (Fig. 4C; pJNK). In addition, we observed that HS+MES treatment up-regulated the level of Hsp72 in the liver of high fat-fed mice (Fig. 4C).

### Treatment with HS+MES decreased fat accumulation in adipose tissues and liver of high fat-fed mice

**Figure 5:**
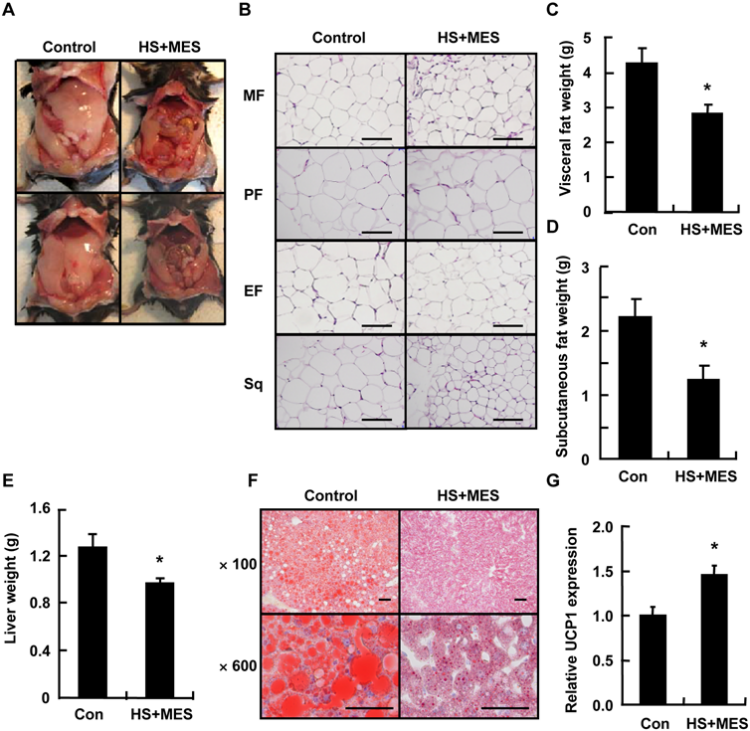
HS+MES decreased the fat content in mice liver and adipose tissues. (A) Ventral aspect of representative high fat-fed mice sham-treated (control) or treated with HS+MES with exposed peritoneal cavity, showing decrease of visible adipose tissues in treated mice. (B) Histological staining with H&E of the visceral fat (mesenteric fat (MF), perinephric fat (PF) and epididymal fat (EF)) and subcutaneous fat (Sq) in high fat-fed control and HS+MES-treated mice. Scale bars, 100 µm. (C and D) Mice were sacrificed after a 15-h fast at the 15^th^ week of treatment, and visceral fat (C) and subcutaneous fat (D) weights were determined (n = 8 per group). (E) Liver weight of control and HS+MES-treated mice was determined. Data are presented as mean±S.E. (n = 8 per group). *P<0.05 assessed by unpaired *t* test. (F) Staining of fat droplets by Oil Red O in liver tissues of control and HS+MES-treated mice. Scale bars, (×100) 200 µm, (×600) 100 µm. (G) Total RNA, extracted from brown adipose tissues of high fat-fed control and HS+MES-treated mice, was subjected to semi-quantitative RT-PCR. Relative amount of UCP1 mRNA was normalized to HPRT (internal control) and quantified using Image Gauge software. Data are presented as mean±S.E. (n = 3 per group). *P<0.05 assessed by unpaired *t* test.

Since treatment with HS+MES, compared with sham-treated control, increased the serum adiponectin level (Fig. 2C, 3E), which is known to be inversely related to the degree of adiposity [16], [31], we investigated whether this treatment decreases adipose tissue in high fat-fed mice. We found a substantial decrease of white adipose tissue mass in HS+MES-treated mice compared with control mice (Fig. 5A). H&E staining showed reduced size of adipocytes in white adipose tissues of the treated mice (Fig. 5B). Visceral fat and subcutaneous fat weights and liver weight were also reduced in these mice (Fig. 5C–5E). Staining with Oil Red O revealed that the lipid droplets were remarkably decreased in the liver of HS+MES-treated mice (Fig. 5F). Moreover, HS+MES significantly increased, in brown adipose tissue, the mRNA expression of uncoupling protein 1 (UCP1; Fig. 5G), which is known to regulate tissue diet-induced thermogenesis [32]. These results signify that HS+MES treatment improved the fat metabolism in high fat-fed mice.

### Treatment with HS+MES ameliorated hyperglycemia in db/db mice

**Figure 6:**
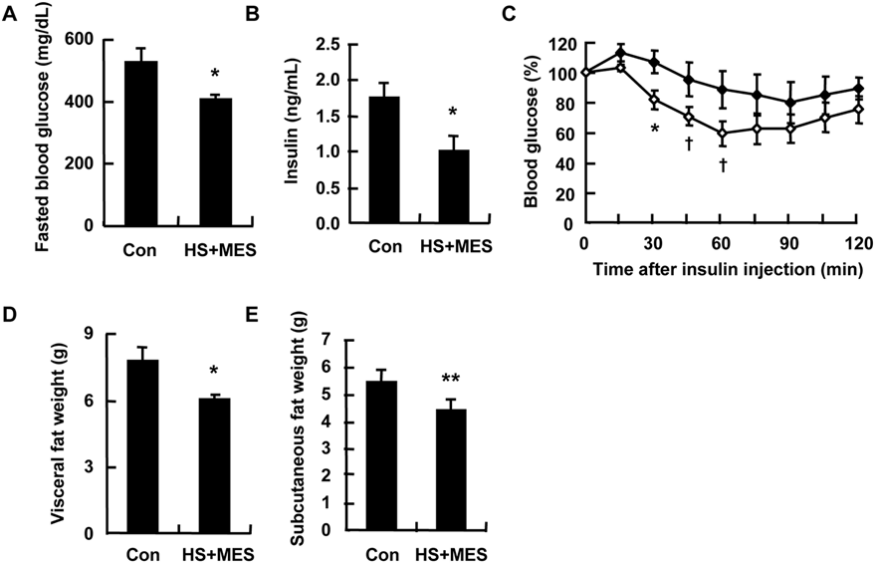
HS+MES ameliorated insulin resistance in db/db mice. (A–C) Blood glucose (A), serum insulin (B) and insulin tolerance (C) of sham-treated or HS+MES-treated db/db mice (n = 5 per group) were measured at the 12^th^ week of treatment in the 15-h (A and B) or 6-h (C) fasted state. Symbols: black diamonds = control; white diamonds = HS+MES. (D and E) Mice were sacrificed after a 15-h fast at the 15^th^ week of HS+MES treatment. Visceral fat (D) and subcutaneous fat (E) weights were determined. Data are presented as mean±S.E. (n = 5 per group). ^†^ *P*<0.1, *P<0.05, **P<0.01 assessed by unpaired *t* test.

To further examine the effect of HS+MES on another mouse model of type 2 diabetes, we used 6-week-old male db/db mice, which exhibit defects in leptin receptor and develop obesity because of hyperphagia and decreased energy expenditure [33]. We used the same condition of treatment as those for the high fat-fed mice. There were no observable differences in body weight and food intake between the HS+MES-treated and sham-treated db/db mice (data not shown). After 12 weeks of HS+MES treatment, fasted blood glucose and serum insulin levels were significantly decreased in treated db/db mice (Fig. 6A,B). Insulin tolerance test suggests that this treatment improved insulin sensitivity in treated db/db mice (Fig. 6C). Moreover, visceral fat and subcutaneous fat weights were significantly decreased in HS+MES-treated mice (Fig. 6D,E). Thus, HS+MES also attenuated insulin resistance associated with obesity and improved fat metabolism in db/db mice.

### HS+MES showed no adverse effects in standard diet-fed mice

**Figure 7:**
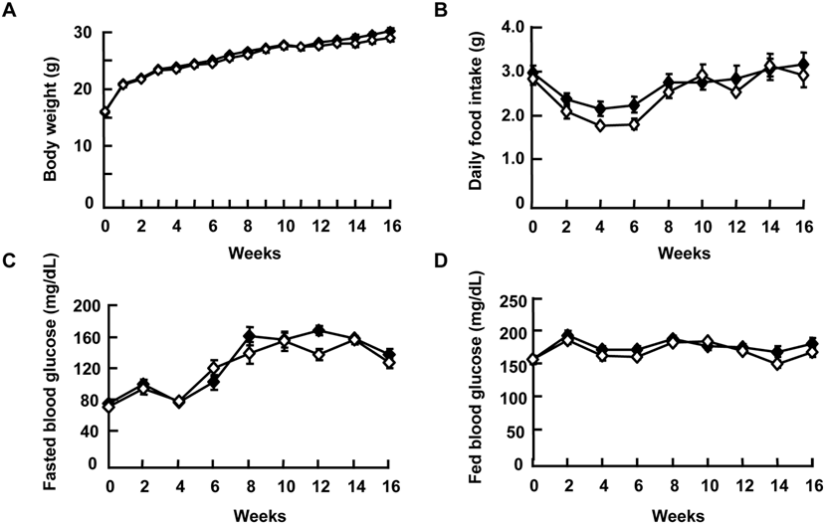
HS+MES had minimal effects on standard diet-fed mice. (A) Body weight of 6-week-old sham-treated (control) and HS+MES-treated mice was measured once a week for 16 weeks during the course of treatment. (B–D) Daily food intake (B), blood glucose levels of fasted (C) and fed mice (D) were measured every 2 weeks during the 16-week treatment. Data are presented as mean±S.E. (n = 5 per group). Symbols: black diamonds = control; white diamonds = HS+MES.

To determine possible adverse effects of HS+MES treatment in normal mice, we confirmed various parameters on standard diet-fed, lean mice. Within 16 weeks of treatment, body weight and food intake did not differ between HS+MES-treated group and control group (Fig. 7A,B). HS+MES for 16 weeks did not induce a difference in the blood glucose levels in standard diet-fed mice compared with sham-treated control when blood glucose was examined under fasted and fed conditions (Fig. 7C,D). These data suggest that HS+MES had no adverse side effects on normal, lean mice.

### MES increased Akt phosphorylation through PI3K in HepG2 cells

**Figure 8:**
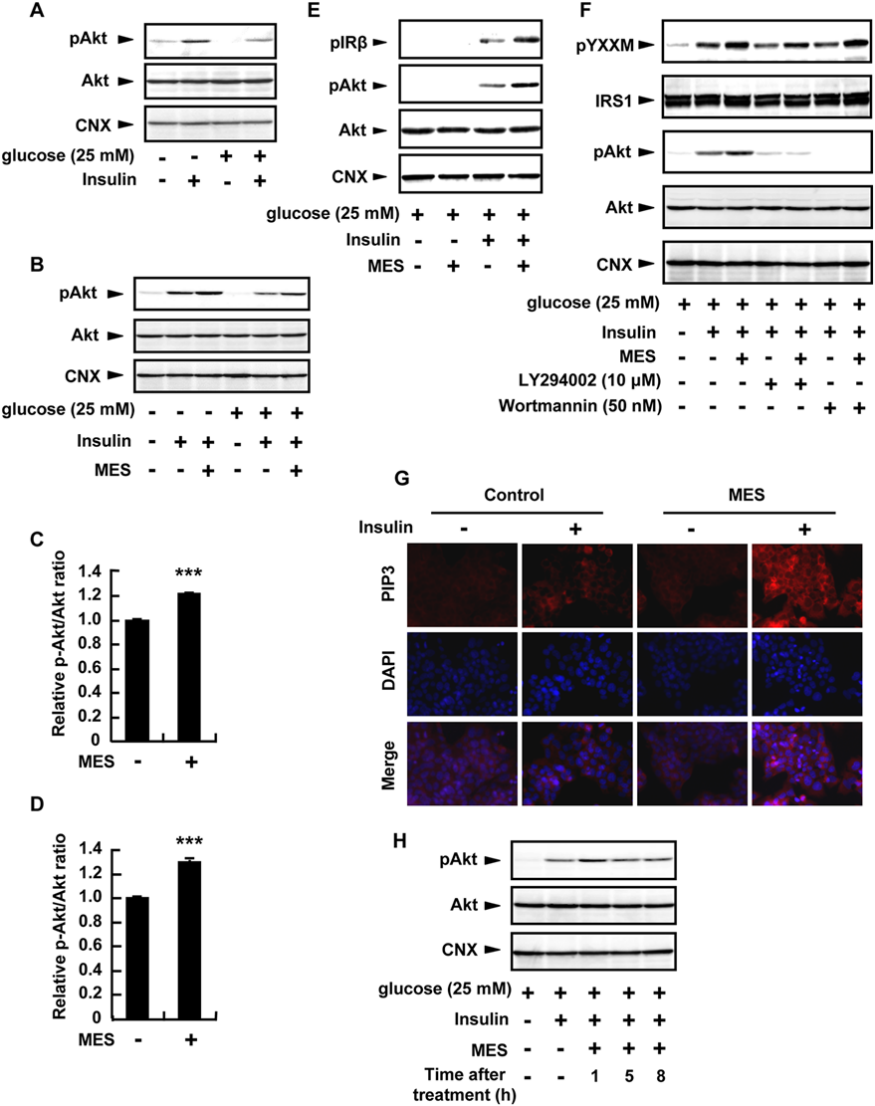
MES increased Akt phosphorylation through PI3K pathway in HepG2 cells. (A) Cells were incubated in medium containing either normal or high glucose concentration and were stimulated with insulin before harvesting. (B) Cells were incubated in medium containing either normal or high glucose concentration and then treated with MES (5 V, 0.1 ms) for 10 min. After 1 h, cells were stimulated with insulin. (C and D) Relative amount of p-Akt was normalized to total Akt in either normal (C) or high (D) glucose concentration, and quantified using Image Gauge software. Data are presented as mean±S.E. (n = 3 per group). ***P<0.001 assessed by unpaired *t* test. (E) Cells were treated with MES for 10 min. After 1 h, cells were stimulated with insulin. (F) Cells were treated with MES for 10 min with or without the PI3K inhibitors, LY294002 and Wortmannin. After 1 h of re-incubation at 37°C, cells were stimulated with insulin. (G) HepG2 cells were treated with MES for 10 min. After 1 h, cells were stimulated with insulin, stained with PIP3 and DAPI, and visualized by fluorescent microscope. Scale bars, 100 µm. (H) Cells were treated with MES for 10 min. After the indicated time of re-incubation at 37°C, cells were stimulated with insulin before protein extraction. For B–H, control cells in these experiments were sham-treated.

To further investigate the effect of MES on Akt phosphorylation, we examined the phosphorylation status of Akt *in vitro* using HepG2 cells. This cell line was previously used to determine the role of PI3K/Akt pathway in insulin signaling and, by incubating in high-glucose medium, was used as *in vitro* model of insulin resistance [34], [35], [36]. Consistent with previous report, HepG2 cells exposed to high glucose concentration (25 mM) exhibited reduced insulin-stimulated Akt phosphorylation (Fig. 8A, [34]). MES (5 V) with pulse duration of 0.1 ms, which did not induce cell toxicity [25], applied for 10 min to normal or high glucose-exposed cells effectively increased the insulin-stimulated phosphorylation of Akt, 1 h after treatment of cells (Fig. 8B–8D). Next, we determined the phosphorylation status of IRβ subunit. MES enhanced the phosphorylation of IRβ consistent with the increase of Akt phosphorylation (Fig. 8E). We also examined the effect of MES on downstream molecules of insulin signaling. As expected, MES promoted the tyrosine phosphorylation of YXXM motif in IRS protein (IRS-1 and IRS-2) (Fig. 8F; pYXXM) that leads to the activation of PI3K and Akt phosphorylation in the liver [37]. Furthermore, we observed that the Akt phosphorylation induced by MES was suppressed in the presence of PI3K inhibitors, LY294002 and wortmannin (Fig. 8F; pAkt). Consistent with these results, PIP3 immunoreactivity was enhanced by MES treatment in insulin-stimulated HepG2 cells compared with sham-treated control (Fig. 8G). We detected that the enhancement of Akt phosphorylation peaked at 1 h after MES treatment and gradually decreased thereafter (Fig. 8H). Taken together, MES affected the activation of insulin signaling molecules, although the enhancement of insulin signaling pathway by MES was sustained for only a short time.

### HS+MES extended the duration of Akt phosphorylation through the increase of Hsp72 expression in HepG2 cells

**Figure 9:**
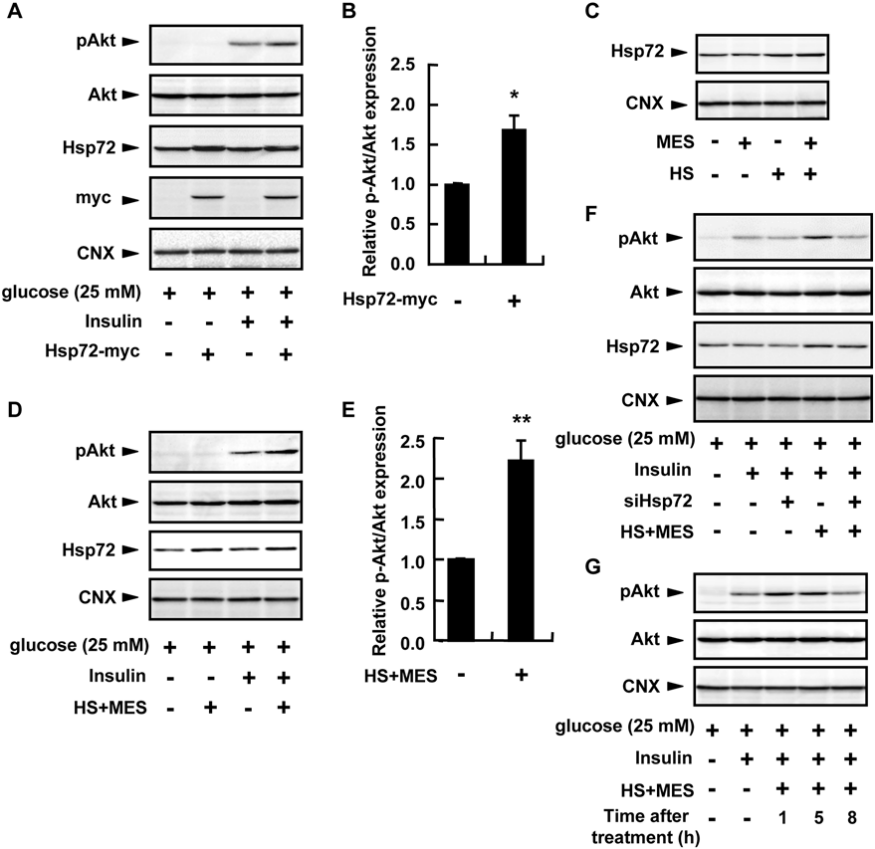
HS+MES promoted the phosphorylation of Akt in HepG2 cells. (A) Cells were transiently transfected with Hsp72-myc plasmid or empty vector. After 24 h, cells were incubated in medium containing high concentration of D-glucose. Before harvesting, cells were stimulated with insulin. (B) Relative amount of p-Akt was normalized to total Akt and quantified using Image Gauge software. Data are presented as mean±S.E. (n = 3 per group). *P<0.05 assessed by unpaired *t* test. (C) Cells were treated with HS and/or MES for 10 min. After 5 h, cell lysates were extracted. (D) Cells were treated with HS+MES for 10 min. After 5 h, cells were stimulated with insulin for 10 min before protein extraction. (E) Relative amount of p-Akt was normalized to total Akt and quantified using Image Gauge software. Data are presented as mean±S.E. (n = 3 per group). **P<0.01 assessed by unpaired *t* test. (F) Cells were transiently transfected with Hsp72 siRNA or GL2 siRNA for 24 h, then incubated in medium containing high concentration of D-glucose for 19 h. Cells were treated with HS+MES for 10 min. After 5 h, cells were stimulated with insulin. (G) Cells were treated with HS+MES for 10 min. After the indicated time of re-incubation at 37°C, cells were stimulated with insulin before protein extraction. For (A, C, D, F, G), lysates were subjected to immunoblotting with the indicated antibodies. For (C–G), control cells were sham-treated.

Insulin-stimulated Akt phosphorylation has also been observed to be up-regulated in Hsp72 transgenic mice [24]. The mechanism of which is most probably through the inhibitory action of Hsp72 on JNK, which plays a role in insulin resistance [20], [22]. To investigate the effect of Hsp72 overexpression, we transfected Hsp72 plasmid in HepG2 cells. The Akt phosphorylation induced by insulin was enhanced upon Hsp72 overexpression (Fig. 9A,B). We next applied HS together with MES to HepG2 cells and determined the Hsp72 expression and the phosphorylation status of Akt. The combination treatment of HS and MES for 10 min increased the Hsp72 expression in protein lysates of HepG2 cells (Fig. 9C) isolated 5 h after treatment. Consequently, this treatment enhanced the insulin-stimulated Akt phosphorylation in HepG2 cells exposed to high glucose similar to Hsp72 overexpression (Fig. 9D,E). Knockdown of Hsp72 by siRNA in these cells reduced the effect of HS+MES (Fig. 9F), suggesting that the effect of HS+MES is partly mediated by Hsp72.

To clarify the action of HS and MES on Akt activation, we determined their time-dependent effect. Cells were treated for 10 min with HS and MES, and lysates were isolated at the indicated time points. The combination of HS and MES induced a sustained enhancement of Akt phosphorylation from 1 h up to 5 h after treatment in insulin-stimulated, high glucose-exposed HepG2 cells (Fig. 9G). These results confirmed that MES activated Akt, and together with HS, which up-regulates Hsp72, could sustain the level of phosphorylated Akt.

## Discussion

Insulin resistance is a crucial risk factor for the development of type 2 diabetes. Although the pathogenesis of insulin resistance is largely unknown, studies have proven that it is due to a defect in insulin signaling [11], [14], [16]. PI3K/Akt pathway is one of the signaling cascades activated by insulin and plays a major role in hepatic glucose homeostasis [35], [38], and Akt knockout mice show insulin resistance that later developed to a phenotype of type 2 diabetes [14]. These studies suggest that defective Akt signaling contributes to the development of insulin resistance. It was previously reported that electrical signals activate PI3K/Akt [8], thus we investigated whether application of low-intensity current could increase Akt phosphorylation and affect insulin signaling. In addition, Hsp72 was also shown previously to alleviate insulin resistance [24]. We showed here that the use of low intensity current together with short-duration HS activated Akt *in vivo* (Fig. 4A) and resulted in higher insulin sensitivity, lower blood glucose level and higher serum adiponectin in high fat-fed mice (Fig. 2–3). HS+MES treatment also led to an improvement of hepatic steatosis (Fig. 5E,F), a condition which has been linked with insulin resistance [39], [40]. The effect of HS+MES on insulin signaling is likely through the capacity of electrical signal to trigger the activation of Akt (Fig. 8, [8]) and of HS to up-regulate Hsp72, which in turn inhibits JNK and activates the insulin signaling pathway (Fig. 4C, Fig. 9 [24], [41]). Although the core body temperature of the mice was less than 42°C, the method of mild HS that we used was enough to induce the expression of Hsp72 (Fig. 4D).

Type 2 diabetes and obesity have characteristics of inflammatory conditions with increased blood levels of pro-inflammatory cytokines, such as interleukin (IL)-6, IL-1 and TNF-α [42], which attenuate insulin receptor signaling [43]. The anti-diabetic agent, pioglitazone, has been reported to decrease TNF-α and increase serum adiponectin in type 2 diabetic patients [44]. Thus, it is notable that the combination treatment of HS and MES significantly decreased serum TNF-α level and increased serum adiponectin in mice chronically fed with high-fat diet (Fig. 3E,F).

One of the observations in this study is that although there was no difference in body weight and food intake between control and treated groups of high fat-fed mice (Fig. 2F,G) and db/db mice, there was a significant decrease in the visceral fat and subcutaneous fat weights in mice treated with the combination of HS and MES (Fig. 5C–D, Fig. 6D–E). This might be due to an increase in the muscle mass of the treated mice, as it has been suggested that heat stress, which increases Hsp72 and calcineurin levels, may cause muscle hypertrophy [45].

Our data indicated that one of the main target tissues of HS+MES is the liver. Other target tissues, such as adipose tissues and skeletal muscles, might also be affected by HS+MES, and are involved in the observed amelioration of the diabetic phenotype. In addition to the increased insulin signaling in the liver with HS+MES treatment, we observed that HS+MES affected the levels of serum TNF-α and adiponectin, which are adipocytokines secreted by adipose tissues (Fig. 3E,F), as mentioned above. HS+MES also enhanced the cell surface translocation in mice skeletal muscle of GLUT4 (Fig. S1), the major regulatory molecule for insulin-stimulated glucose uptake in skeletal muscles, indicating an improved response to insulin stimulation in skeletal muscles. These data suggest that HS+MES may directly affect major insulin target tissues or may exert an anti-diabetic effect in one tissue, leading to secondary effects in the other tissues.

Low-intensity current as well as heat shock have been applied as treatment modalities in clinical setting for a range of diseases with relatively few side effects [6], [46]. The absence of observable adverse effects of HS+MES treatment in normal mice (Fig. 7) in our study suggests its safety, although it is necessary to assess the long-term effects of this treatment. Due to technical limitations we could not measure the extent of electrical conductance in cells and tissues. Electrotherapeutic units of low voltage may produce currents of intensities up to a few microamperes and milliamperes, but measuring the current distribution of an applied electric current in biological tissues is hampered by several factors. Electrical charges in tissues are transferred by multiple mechanisms such as the migration of ions, membrane capacitance, and rotation of polar molecules [47], [48], [49]. Moreover, these electrical properties vary between tissues. Different cell types show subtly different responses to direct current electrical field due to variable local tissue resistances, the extracellular matrix composition, the coexistence of growth factors and neurotransmitters, and the level of second messengers within the cells [50]. Because low-intensity current and heat shock may have an impact on many cellular processes and functions, we cannot rule out the possibility that signaling pathways, other than the PI3K/Akt pathway, are affected by HS+MES treatment to produce the observed effects. Further investigation on the possible regulation of other signaling molecules by HS+MES may be necessary to provide deeper mechanistic insight to the effects of HS+MES. In conclusion, we showed here that the combination of HS and MES treatment ameliorated insulin resistance and decreased fat accumulation in diabetes mouse models, in part, by enhancing Akt activation.

## Supporting Information

Figure S1 (0.34 MB PDF)
