# Supplementary material for: Mild Electrical Stimulation with Heat Shock Ameliorates Insulin Resistance via Enhanced Insulin Signaling
Source: PLoS One. 2008 Dec 30;3(12):e4068. doi: 10.1371/journal.pone.0004068 (PMC2603588; doi:10.1371/journal.pone.0004068)
Supplement: Figure S1 — (0.34 MB PDF) [file pone.0004068.s001.pdf]

Figure S1

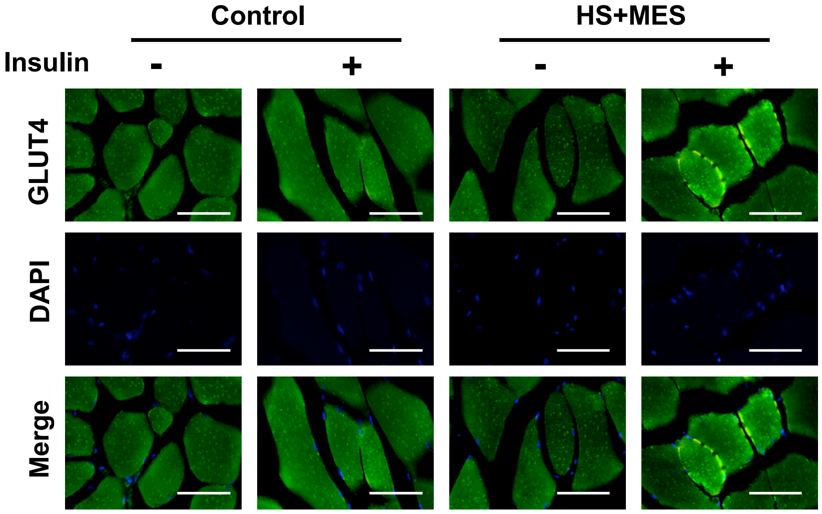

Figure S1. Skeletal muscle tissues, isolated at the 15th week after initiation of treatment from high fat-fed control or HS + MES-treated mice with or without 5 units of insulin stimulation through inferior vena cava, were dissected in frozen sections and stained with GLUT4 and DAPI in the same manner as described in Methods. The stained sections were visualized by fluorescent microscope. Scales bars, 100 μm
